# A survey of current state of training of plastic surgery residents

**DOI:** 10.1186/s13104-017-2561-5

**Published:** 2017-06-27

**Authors:** Asra Hashmi, Faraz A. Khan, Floyd Herman, Nathan Narasimhan, Shaher Khan, Carrie Kubiak, Eti Gursel, David A. Edelman

**Affiliations:** 1 Department of Plastic Surgery, Wayne State University/Detroit Medical Center, Detroit, USA; 2 Department of General Surgery, Wayne State University/Detroit Medical Center, Detroit, USA; 3 Department of Surgery, Wayne State University/Detroit Medical Center, Detroit, USA; 4 4160 John R Street, Detroit, 48201 MI USA

**Keywords:** Training, Residency, Survey, Quality, Plastic surgery

## Abstract

**Background:**

Plastic surgery training is undergoing major changes however there is paucity of data detailing the current state of training as perceived by plastic surgical trainees. Our aim was to determine the quality of training as perceived by the current trainee pool and their future plans.

**Methods:**

A 25-item anonymous survey with three discrete sections (demographics, quality of training, and post-graduate career plans) was developed and distributed to plastic surgery residents during the academic year 2013. With the confidence interval of 95% and margin of error of 10%, our target response rate was 87 responders.

**Results:**

We received a total of 114 respondents with all levels of Post Graduate Year in training represented. Upon comparison of residents with debt of <100,000 to residents with a debt of >250,000, those with higher debt were significantly less interested in fellowship training (p value 0.05) and were more likely to pursue private practice (p value <0.01). Disciplines within plastic surgery least offered as a separate rotation were microsurgery (45%) followed by aesthetic surgery (33%). 53.7% of the residents felt that they were least trained in aesthetic surgery followed by burn surgery 45.4%. Of note 56.4% intended to seek additional training after residency. Moreover residents with an average of 6.4 months of experience in an individual subspecialty were more likely to feel comfortable with that specialty.

**Conclusions:**

This survey highlights the areas and subspecialties that deserve attention as perceived by the current trainee pool.

**Electronic supplementary material:**

The online version of this article (doi:10.1186/s13104-017-2561-5) contains supplementary material, which is available to authorized users.

## Background

Training in plastic surgery is undergoing major changes including a transition from independent to integrated models of training with a parallel evolution in subspecialty training. The American Council of Academic Plastic Surgeons describes the two pathways into plastic surgical training. The Independent model consists of 2–3 years of formal training in plastic surgery following satisfactory completion a formal training program in general surgery, otolaryngology, neurosurgery, orthopedic surgery, urology, or oral and maxillofacial surgery. Whereas training in the Integrated model requires 5 or 6 years of accredited residency under the authority and direction of the plastic surgery program director. Previous investigators have analyzed the fields of microsurgery, craniofacial surgery and aesthetic surgery individually in an attempt to predict the future course of the field of plastic surgery [1–6]. Other studies compared integrated versus independent models of plastic surgical training however were unable to allude to any significant impact of the model of training on the individual resident’s professional career and choices [7]. Although these studies provide valuable insight, most concentrate on a specific subspecialty or are reliant on data from a single program. We therefore designed a national survey of all US plastic surgery residents with three main objectives: Firstly, to define the demographic characteristics of the plastic surgery residents to better understand the population under study, secondly, to assess how the plastic surgery residents perceive the current state of training and finally, to delineate their future goals.

## Methods

After approval by the Wayne State University institutional review board, a 25-item anonymous online survey (SurveyMonkey) was developed. To ensure reliability ‘alternate form’ questions were included in the questionnaire. Additionally to maximize validity some basic measures were taken for e.g., some questions were designated ‘required’ and a requirement of minimum number of answers was established. The survey was electronically distributed to all plastic surgery training programs recognized by the Accreditation Council for Graduate Medical Education (ACGME) during the 2013 calendar year. This process was repeated three times and covered all plastic surgery training programs in the United States. Survey comprised of three discrete sections focusing on demographics, quality of training, and post-graduate career plans. Descriptive statistics including means for continuous variables and frequency tables for categorical variables were generated. Using a confidence interval of 95% and a 10% margin of error target response rate of 87 responders was calculated. Due to an insufficient number of responses for questions 23 and 25 of the questionnaire (Additional file 1) were excluded from the analysis and discussion.

## Results

### Demographics

**Fig. 1:**
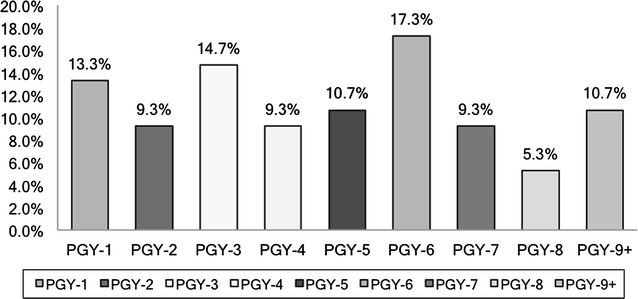
Postgraduate level of training

There were 114 responders to the survey giving us a survey response rate of 12.7%. 86% of the responses were obtained in the month of June, at the end of the academic cycle 2013, 14% responses were obtained from July through October 2013. All levels of Post Graduate Year (PGY) in training were represented (see Fig. 1).

**Fig. 2:**
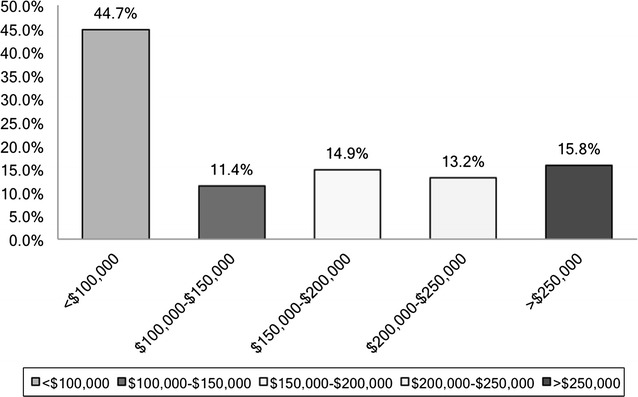
Educational debt

55.3% of trainees reported educational debt in excess of $100,000 where as 44.7% trainees had educational debt less that $100,000 (see Fig. 2). Moreover among senior residents (PGY 4–9) educational debt was reported to be in excess of $100,000 by 57.5% respondents. 50.6% residents in the integrated had education related debt of >$100,000 in contrast to 65.7% of independent track residents.

### Quality of training

**Fig. 3:**
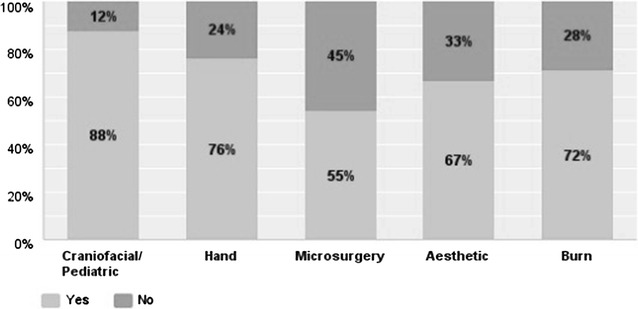
Rotations provided in subspecialties

**Fig. 4:**
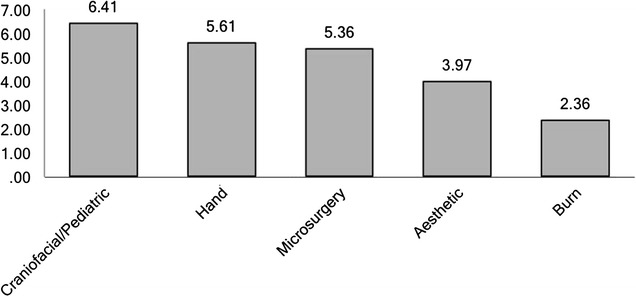
Months spent in subspecialty training

**Fig. 5:**
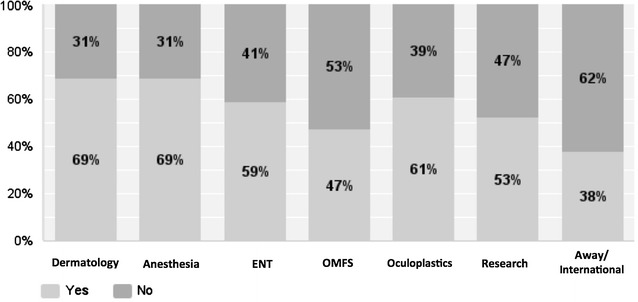
Elective rotation offered

**Fig. 6:**
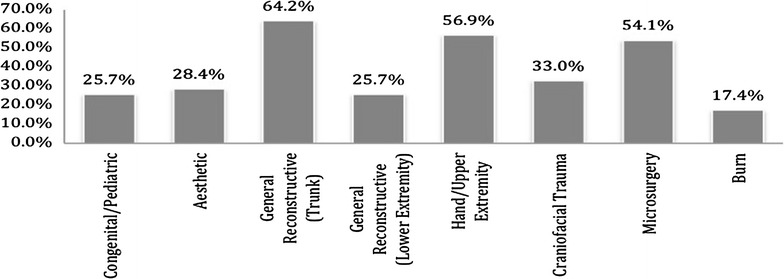
Areas in which most training is provided by programs

**Fig. 7:**
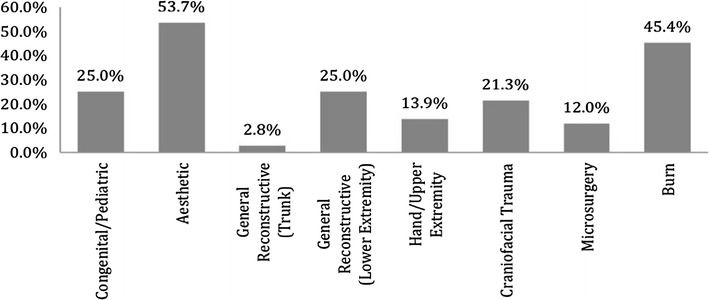
Areas in which least training is provided by programs

Disciplines in plastic surgery least offered as a separate rotation were microsurgery (45%) followed by aesthetic surgery (33%) (see Fig. 3). With respect to subspecialty experience, participants reported spending most amount of time in craniofacial surgery (6.4 months) followed by hand surgery (5.6 months) (see Fig. 4). Residents reported dermatology (69%), anesthesia (69%) and oculoplastics (61%) as the most commonly offered elective rotations (see Fig. 5). 64.2% of the responders felt that they were most extensively trained in general reconstructive procedures of the trunk (see Fig. 6). When residents were asked to specify specialties they were least trained in; aesthetic surgery was the most commonly reported specialty (53.7%) followed by burn surgery (45.4%) (see Fig. 7).

**Fig. 8:**
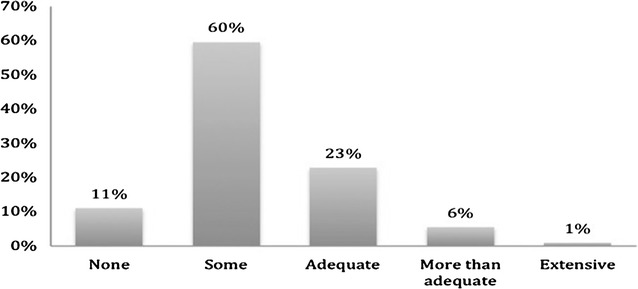
Practice management training

Residents who self-reported adequate training in a specialty on average spent a longer duration of time rotating in that specialty as compared to those that felt that they were least trained in the same specialty. (Aesthetic surgery 6 months versus 3 months; p value <0.01, burn surgery 6 months versus 1 month, p value <0.01, craniofacial 7 months versus 4 months, p value <0.01, hand surgery 7 months versus 3 months, p value 0.02; microsurgery 6 months versus 4 months, p value 0.3) (Table 1). Exposure to practice management reported by trainees is shown in Fig. 8.

**Table 1:** Average number of months spent in subspecialties to feel most and least trained in

	Most trained Mean ± SD	Least trained Mean ± SD	p value (<0.05)
Aesthetic surgery	6 ± 3.85	3 ± 1.59	0.0001
Microsurgery	6 ± 5.4	4 ± 3.63	0.3
Burn surgery	6 ± 8.34	1 ± 0.81	0.0005
Craniofacial	7 ± 3.54	4 ± 1.94	0.001
Hand	7 ± 3.87	3 ± 2.18	0.02

### Career plans

Increasing debt (100,000, 100–250 and >250 k) was associated with a trend towards decreased inclination to pursue fellowship training (65.3, 54.5 and 35.3% respectively (p value 0.17) (Table 2). When asked about their preferred type of practice, 26.67% of residents in <100,000 debt group, 70% of residents in 100,000–250,000 group and 81.8% of residents in >250,000 debt group were interested in private practice (p value 0.02). In contrast 33.3% of residents in <100,000 debt group, 15% of residents in 100,000–250,000 group and 18.2% of residents in >250,000 debt group, were interested in academic practice (0.66). On second level of analysis, residents with a debt of <100,000 were compared to residents with a debt of >250,000, interest in additional fellowship (p value 0.05) and interest in private practice (p value <0.01) reached statistical significance for this comparison in our study.

**Table 2:** Career plans and educational debt

	<100,000 (N = 51)	100,000–250,000 (N = 45)	>250,000 (N = 18)	p value
Interest in additional fellowships	65.3% (32)	54.5% (24)	35.3% (6)	0.17
Interest in private practice	26.67% (4)	70% (14)	81.8% (9)	0.02
Interest in academic practice	33% (5)	15% (3)	18.2% (2)	0.66
Interest in hospital employed nonacademic practice	40% (6)	15% (3)	0% (0)	0.15

In the integrated resident group 71.4% were interested in pursuing additional fellowships as opposed to independent resident group in which only 21.2% were interested in additional fellowship training (p value <0.01) (Table 3). However only 4.76% of the integrated residents were interested in academic practice versus 36% of independent residents who showed an interest in academia (p value <0.01). There was no statistically significant association of gender with interest in fellowship training or type of practice (Table 4).

**Table 3:** Career plans and type of training

	Interest in additional fellowships	Interest in starting practice	Interest in academia	Nonacademic hospital employed	Private practice
Integrated residents % (N = 77)	71.4 (55)	28.57 (22)	4.76 (1)	33.3 (7)	61.9 (11)
Independent residents % (N = 33)	21.2 (7)	78.8 (26)	36 (9)	8 (2)	56 (14)
p value (<0.05)	0.000003	0.000003	0.02	0.07	0.9

**Table 4:** Career plans and gender

	Interest in additional fellowships	Interest in starting practice	Interest in academia	Nonacademic hospital employed	Private practice
Male residents % (N = 80)	54.5 (42)	45.5 (35)	21.2 (7)	24.2 (8)	54.4 (18)
Female residents % (N = 34)	60 (20)	40 (13)	23 (3)	7.6 (1)	69.1 (9)
p value (<0.05)	0.67	0.73	0.86	0.43	0.43

**Fig. 9:**
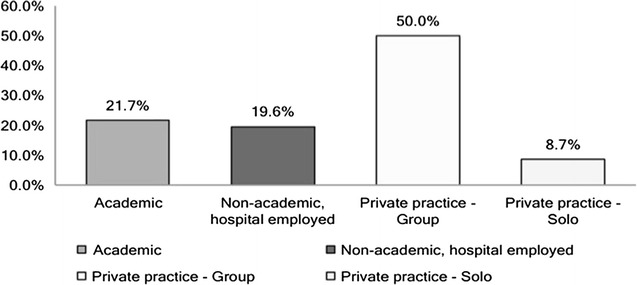
For those planning on seeking employment the kind of practice trainees wished to pursue

**Fig. 10:**
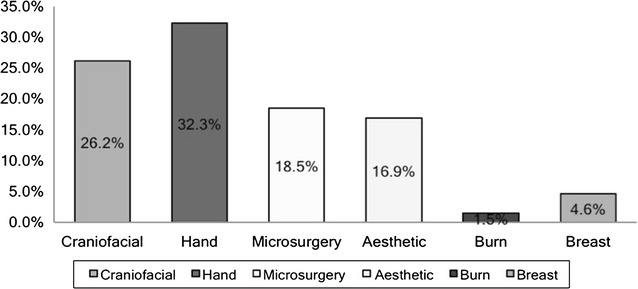
For those seeking additional training, the specialization trainees wished to pursue

For those seeking employment 50% wished to go into private group practice, 21.7% were interested in pursuing an academic career and 8.7% expressed interest in starting out as private solo practice (see Fig. 9). 56.4% responders intended to seek additional training after residency. Among these 32.3% were interested in hand surgery and 26.2% showed interest in craniofacial surgery followed by 18.5% who were interested in microsurgery (Fig. 10).

For residents interested in pursuing a craniofacial fellowship 88.3% were training in an integrated program, the average time spent on a craniofacial rotation in residency was 6 months and average time spent in research was 22 weeks. For those interested in aesthetic surgery fellowship, 72.7% were from an integrated program, spent an average of 5 months in aesthetic surgery and an average of 4 weeks in research. For those interested in microsurgery, 83% were from integrated programs, spent an average of 10 months in microsurgery during the course of their training and spent an average of 18 weeks in research. For residents interested in hand surgery fellowship, 85.7% were from integrated program, average months spent in hand surgery rotation were 5 months and average weeks spent in research were 13 weeks (Table 5). Questionnaire is also included for reference (Additional file 1).

**Table 5:** Type of training, months spent in respective specialty and research time spent by fellowship candidates

	Interest in craniofacial surgery (n = 17)	Interest in aesthetic surgery (N = 11)	Interest in microsurgery (N = 12)	Interest in hand surgery (N = 21)
Integrated residents	88.3% (15)	72.7% (8)	83% (10)	85.7% (18)
Average months spent in specialty during training	6	5	10	5
Average weeks spent in research during training	22	4	18	13

## Discussion

Providing adequate training in plastic and reconstructive surgery is fraught with several challenges and this survey was performed to investigate the various shortcomings in training perceived by the trainees.

First, we attempted to define the demographics of the population under study and change in the behavior with certain demographic characteristics including education debt. One of the findings that stood out was that educational debt exceeded $100,000 in more than half of the trainees. Upon analyzing senior residents (PGY 4–9) separately educational debt still exceeded 100,000 in more than half of senior residents. Imahara et al. reported that outstanding educational debt does not influence career plans of trainees [8]. However, that study was limited by a small sample size; the highest amount of debt that they looked at was 100,000 and authors of that study neglected to further categorize the group with a debt of >100,000. We analyzed the population with a debt ranging from <100,000 to >250,000 and divided them in increments of 50,000 and then compared them based on type of training and career goals. In our study independent residents had a higher debt compared to the integrated group of residents. We also noticed a trend towards declining interest in fellowship training and academic career with incremental amount of educational debt. However, there was a statistically significant enhanced interest in pursuing private practice among those reporting higher amount of debt. Moreover in subgroup analysis, upon comparison of residents with debt of <100,000 to residents with a debt of >250,000, residents with higher debt were significantly less interested in fellowship training (p value 0.05) and were found to be more interested in private practice (p value <0.01) at the conclusion of their training.

Second, we found that career plans and goals do not vary by gender. As the number of women pursuing a medical career in surgery continues to increase [9, 10], it is interesting to note that that women’s goals and aspirations do not significantly differ from men. In our study interest in fellowship training as well as academic, private or hospital-based practice were similar between men and women.

Our third interesting observation was related to quality of training. Our findings validate the common perception that comfort level in subspecialty training is a product of experience and therefore time spent in the respective specialty. For all specialties combined, in our study this averaged out to be 6.4 months for the residents who felt comfortable with their training versus 3 months for the residents who felt that they were least trained in the respective specialty. We looked at craniofacial, burn, hand, aesthetic and microsurgery. Statistical significance was reached for all specialties except microsurgery training (Table 1). Some minor observations related to quality of training that also deserve attention are as follows: 45% of responders of our survey did not have a microsurgery rotation. A plausible explanation of this finding maybe that some programs are structured such that microsurgical cases are simply mixed into the daily caseload without offering a specific rotation. For the programs that are indeed deficient in microsurgical training, simulator training maybe a useful alternative [11, 12]. 33% responders also indicated that they lacked an aesthetic rotation in their training program, which also explains the observation that 53.7% trainees felt that they were least trained in aesthetic surgery. Momeni et al. analyzed the quality of aesthetic surgery training in Germany to investigate how these challenges were met abroad [5]. Their study revealed that problems in providing adequate cosmetic surgery training were a product of three factors i.e., lack of curriculum, private patient population demanding to be operated upon by attending physicians only, dearth of cosmetic surgical procedures at major academic centers. Oni et al. suggested some steps to improve quality of aesthetic surgery training. Authors recommended establishment of senior resident cosmetic clinic, cosmetic surgery rotation including outreach programs to include community plastic surgeons for programs weak in cosmetic surgery, inclusion of online education modules and encouragement to attend national meetings [13].

Fourth, we found that that most residents who are interested in fellowship training are from integrated programs (p value <0.01). In retrospect it may be related to higher educational debt that independent residents carry compared to integrated residents. Residents who reported interest in subspecialty training spent an average of 6, 5, 10 and 5 months in craniofacial, aesthetic, microsurgery and hand surgery respectively during the course of their training. This averages out to be 6.5 months for all specialties combined. Moreover residents with an interest in fellowship training spent an average of 14 weeks in research during the course of their training. Despite the extensive amount of time spent in research we noticed a declining interest in academia. We observed that even though nearly half of the graduates intended to pursue a subspecialty fellowship only one-fifth intended to enter academic career, which is consistent with prior smaller studies [4]. Grewal et al. observed that even though a majority of fellowship applicants indicated an aspiration to practice academic medicine, only one-third remained in full-time academics 5 years after the completion of their subspecialty training [14]. Economic constraints developing as a result of rising health care costs in the United States pose significant challenges for and threats to the survival of academic plastic surgery [15].

Fifth, majority of graduating plastic surgery trainees’ complete residency with inadequate business skills as evidenced by this survey. The ever increasing complexity of the United States healthcare system in conjunction with the competitiveness of the plastic surgery marketplace, demand that residencies begin to address practice development in their training of residents.

Findings of this study should be considered in light of potential study limitations, which are as follows: our online survey did not go through vigorous validation process but rather was based on group consensus. However, we used ‘alternate form’ questions in the questionnaire to ensure reliability. Second, there is a potential for response bias in our study because of nature of the topics addressed in our study and also data was subjectively reported.

The study has several strengths that support the validity of findings and suggest potential for future analyses. First, while there have been surveys of graduating residents [8], to our knowledge data regarding demographics that lead to change in behavior pattern including career plans has not been studied. Second, the survey was structured so that most responses were obtained in the last month of academic cycle 2013 giving junior residents an opportunity to gain experience in plastic surgery prior to responding to the survey. Third, scope of our study including representation from all types and years of training as well as from programs all across US offers a comprehensive national picture of plastic surgery residency training.

## Conclusions

This descriptive study presents an overview of resident’s views towards complex, interrelated aspects of residency training including strains and concerns regarding training experience, interest in subspecialty training as well as motivation for the choice of surgical career. Educational debt appears to play in integral role in the choices that are made by residents and thus counseling as well as financial planning programs may aid in preventing bright minds from shying away from pursuing fellowship training as well as an academic career. Furthermore our study highlights that at least 6 months of training in individual subspecialties is necessary in order for residents to get comfortable with the specialty. Findings of our study maybe used to target areas of deficiencies and to design a comprehensive curriculum to improve upon areas of weaknesses. Future analysis should explore ways to improve aesthetic and microsurgery training experience for residents using longitudinal data. We hope that findings of our study will assist programs in improving the quality of plastic and reconstructive surgery training.

## Additional file

**Additional file 1.** Questionnaire.

## Abbreviation

ACGME: Accreditation Council for Graduate Medical Education

## References

[1] Messaoudi T, Bodin F, Hidalgo Diaz JJ, Ichihara S, Fikry T, Lacreuse I, Liverneaux P, Facca S (2015). Evaluation of a new eLearning platform for distance teaching of microsurgery. Chir Main.

[2] Christensen TJ, Anding W, Shin AY, Bishop AT, Moran SL (2015). The influence of microsurgical training on the practice of hand surgeons. J Reconstr Microsurg.

[3] Abdelrahman M (2015). The microsurgery fellowship at Chang Gung Memorial Hospital: blossom of caterpillars. Plast Reconstr Surg Glob Open.

[4] Patel N, Dittakasem K, Fearon JA (2015). Craniofacial fellowship training: where are we now?. Plast Reconstr Surg.

[5] Momeni A, Goerke SM, Bannasch H (2013). The quality of aesthetic surgery training in plastic surgery residency: a survey among residents in Germany. Ann Plast Surg.

[6] Chaput B, Bertheuil N, Jacques J, Smilevitch D, Bekara F, Soler P, Garrido I, Herlin C, Grolleau JL (2015). Professional burnout among plastic surgery residents: can it be prevented? Outcomes of a national survey. Ann Plast Surg.

[7] Roostaeian J, Fan KL, Sorice S, Tabit CJ, Liao E, Rahgozar P, Tanna N, Bradley JP (2012). Evaluation of plastic surgery training programs: integrated/combined versus independent. Plast Reconstr Surg.

[8] Imahara SD, Scott JR, Neligan PC (2009). Career plans of graduating plastic surgery trainees in 2009: the impact of an uncertain economic climate. Plast Reconstr Surg.

[9] Association of American Medical Colleges. Diversity in Medical Education: Facts and Figures 2008. Washington, DC: American Association of Medical Colleges. 2008. https://services.aamc.org/publications. Accessed 26 May 2009.

[10] Physician Specialty Data. A chart book. Association of American Medical Colleges. Washington, DC: Association of American Medical Colleges. 2006. https://services.aamc.org/publications. Accessed 26 May 2009.

[11] Liverneaux PA, Hendriks S, Selber JC (2013). Robotically assisted microsurgery: development of basic skills course. Arch Plast Surg.

[12] Carey JN, Rommer E, Sheckter C (2014). Simulation of plastic surgery and microvascular procedures using perfused fresh human cadavers. J Plast Reconstr Aesthet Surg.

[13] Oni G, Ahmad J, Zins JE (2011). Cosmetic surgery training in plastic surgery residency programs in the United States: how have we progressed in the last three years?. Aesthet Surg J.

[14] Grewal NS, Spoon DB, Kawamoto HK (2008). Predictive factors in identifying subspecialty fellowship applicants who will have academic practices. Plast Reconstr Surg.

[15] Miller SH (1998). Competitive forces and academic plastic surgery. Plast Reconstr Surg.

