# Supplementary material for: A survey of current state of training of plastic surgery residents
Source: BMC Res Notes. 2017 Jun 27;10:234. doi: 10.1186/s13104-017-2561-5 (PMC5488360; doi:10.1186/s13104-017-2561-5)
Supplement: Supplementary file 1 — Additional file 1. Questionnaire. [file 13104_2017_2561_MOESM1_ESM.docx]

**A Survey of Current State of Training of Plastic Surgery Residents**

1. Age of the participant ­­­­­­­­­­­­­­­­­_______ (years)
2. Gender □ Male □ Female
3. Residency Type □ Integrated (6 years combined)

□Independent (3-year fellowship)

1. Post Graduate Year □ PGY 1 □ PGY 2 □ PGY 3 □ PGY 4 □ PGY 5

□ PGY 6 □ PGY 7 □ PGY 8 □ PGY 9+

1. If in an independent program, what was you prior training?

□ General Surgery □ ENT □ OMFS

Other_____________ (please specify)

1. Educational debt □ <100,000

□100,000-150,000

□150,000-200,000

□200,000-250,000

□>250,000

1. Marital Status □ Single □ Married □ Divorced □ Other
2. Number of Children □ 0 □ 1 □ 2 □ 3

□ 4 □ 5 or more

1. Total number of residents in program (including all years)

□ 1-3 □ 4-6 □ 7-9 □ 10-12

□ 12+

1. Number of faculty at sponsoring institution?

□ 1-3 □ 4-6 □ 7-9 □ 10-12

□ 12+

1. Do you have rotations (min 1 month) focused on specific areas of plastic surgery?

Craniofacial/Pediatric □ Yes □No Hand □ Yes □No

Microsurgery □ Yes □No Aesthetic □ Yes □No

Burn □ Yes □No

1. If yes, how many months during your training?

Craniofacial/Pediatric ___________ Hand ___________

Microsurgery ___________ Aesthetic ___________

Burn ___________

1. Does your program have elective rotations?

Dermatology □ Yes □No Anesthesia □ Yes □No

ENT □ Yes □No OMFS □ Yes □No

Oculoplastics □ Yes □No Plastics □ Yes □No

Research □ Yes □No Away/International □ Yes □No

1. If yes, how many weeks do you spend on each rotation?

Dermatology ___________ Anesthesia ___________

ENT ___________ OMFS ___________

Oculoplastics ___________ Plastics ___________

Research ___________ Away/International ___________

1. What areas do you feel your program provides the most training? (Max 3 answers)

□ Congenital/Pediatric □Aesthetic □ General Reconstructive (Trunk)

□ General Reconstructive (Lower extremity) □ Hand/Upper Extremity

□ Craniofacial Trauma □Microsurgery □ Burn

1. What areas do you feel your program provides the least training? (Max 3 answers)

□ Congenital/Pediatric □Aesthetic □General Reconstructive (Trunk)

□ General Reconstructive (Lower extremity) □ Hand/Upper Extremity

□Craniofacial Trauma □Microsurgery □Burn

1. For elective cases you scrub in on, how often do you:

Rarely Sometimes Half of the time Often Almost always

a) Evaluate the patient □ □ □ □ □

in clinic/office preoperatively

b) See the patient at first □ □ □ □ □

at first follow-up

c) See the patient at more □ □ □ □ □

then 1 follow-up

1. How many hours per week are set aside for didactic conference?

□0 □1 □2 □3 □4 □5+

1. What materials are most often used during didactic conferences? (max 3 answers)

□ Inservice questions □Corequest modules □PSEN modules

□Resident presentations □Faculty prepared presentations

□Clinical case scenarios □ Other _________________ (Specify)

1. How much education are you provided regarding practice management (i.e. billing, coding, contract negotiation, insurance participation, practice development, etc.)

□ None □Some □Adequate □More than adequate □Extensive

1. Following completion of your training, do you plan on:

□ Job/ Practice □ Additional fellowship training

1. If planning on seeking employment, what kind of practice will you pursue?

□ Academic □ Non-academic, hospital employed

□ Private Practice-Group □ Private Practice-Solo

1. Primary motivation for pursuing employment?

□ Age □Paying back education debt □Length of training

□Family obligations □Desire to start professional career □Desire to make money □Other

1. If planning on additional training, what specialization will you pursue?

□ Craniofacial □ Hand □ Microsurgery

□ Aesthetic □ Burn □ Breast

□ Other_________ (other specify)

1. Primary motivation for pursuing additional training?

□ Interest in specialization □Insufficient training □Required for practice/employment

□Marketing □Other ­­­­­­­___________ (Other specify)
